# Understanding the medical complexity of children and young people with life-limiting conditions in Wales using linked, routinely collected healthcare data

**DOI:** 10.23889/ijpds.v8i2.2344

**Published:** 2023-09-14

**Authors:** Stuart Jarvis, Andre Bedendo, Lorna Fraser

**Affiliations:** 1 University of York, York, United Kingdom; 2 King's College London, London, United Kingdom

## Objectives

To better understand the complexity and healthcare needs of children and young people in Wales with life-limiting or life threatening conditions to better plan and target healthcare services. Previous attempts to quantify complexity have required primary data collection; this is not feasible at scale, use of existing data is preferred.

## Methods

Routinely collected healthcare and administrative data were linked: primary care data, hospital care data sets, cancer and congenital anomaly registries, paediatric intensive care audit data and death records. Children and young people with life-limiting conditions were identified using a previously developed diagnostic framework. Previous work on conceptualising medical complexity across eight domains was operationalised for the first time using the wide range of available data, with scores across five domains and a total complexity score. The relationship between the complexity score, healthcare use, stage of condition and category of condition was explored.

## Results

Children and young people with life-limiting conditions showed the full range of medical complexity scores, from zero to five, with distributions varying across age groups with increasing complexity at greater ages. Distributions also varied across categories of condition, with congenital and oncology conditions, although among the most prevalent, exhibiting lower medical complexity. Nonetheless, all conditions showed a range of complexities – there were no conditions for which all individuals were either high or low complexity. Complexity scores were correlated with stage of condition and healthcare use and may be used to identify groups likely to have higher healthcare demand or greater risk of clinical instability. While life-limiting conditions were more prevalent in areas of higher deprivation, there was no association between deprivation and medical complexity.

## Conclusion

Assessment of medical complexity from routinely-collected data can be useful in better understanding a population and in targeting and planning care, without requiring additional data collection. This can help to design resilient services that prepare for changing needs and aid targeting of limited resources.

